# Neuroinflammation and Oxidative Stress in Psychosis and Psychosis Risk

**DOI:** 10.3390/ijms18030651

**Published:** 2017-03-17

**Authors:** Henry Barron, Sina Hafizi, Ana C. Andreazza, Romina Mizrahi

**Affiliations:** 1 Research Imaging Centre, Centre for Addiction and Mental Health, Toronto, ON M5T 1R8, Canada; henry.barron1@gmail.com (H.B.); sina.hafizi@camhpet.ca (S.H.); 2 Department of Pharmacology and Toxicology, University of Toronto, Toronto, ON M5S 1A8, Canada; ana.andreazza@utoronto.ca; 3 Campbell Family Mental Health Research Institute, Centre for Addiction and Mental Health, Toronto, ON M5T 1R8, Canada; 4 Department of Psychiatry, University of Toronto, Toronto, ON M5T 1R8, Canada

**Keywords:** schizophrenia, psychosis, neuroinflammation, oxidative stress

## Abstract

Although our understanding of psychotic disorders has advanced substantially in the past few decades, very little has changed in the standard of care for these illnesses since the development of atypical anti-psychotics in the 1990s. Here, we integrate new insights into the pathophysiology with the increasing interest in early detection and prevention. First, we explore the role of *N*-methyl-d-aspartate receptors in a subpopulation of cortical parvalbumin-containing interneurons (PVIs). Postmortem and preclinical data has implicated these neurons in the positive and negative symptoms, as well as the cognitive dysfunction present in schizophrenia. These neurons also appear to be sensitive to inflammation and oxidative stress during the perinatal and peripubertal periods, which may be mediated in large part by aberrant synaptic pruning. After exploring some of the molecular mechanisms through which neuroinflammation and oxidative stress are thought to exert their effects, we highlight the progress that has been made in identifying psychosis prior to onset through the identification of individuals at clinical high risk for psychosis (CHR). By combining our understanding of psychosis pathogenesis with the increasing characterization of endophenotypes that precede frank psychosis, it may be possible to identify patients before they present with psychosis and intervene to reduce the burden of the disease to both patients and families.

## 1. Introduction

Schizophrenia is a debilitating mental disorder that affects about one percent of the population. It is characterized by positive symptoms (e.g., abnormal perceptions and beliefs), negative symptoms (e.g., anhedonia and social withdrawal) and cognitive deficits. It is believed to be multifactorial and heterogeneous in its etiology, such that multiple pathological processes converge on a cluster of affiliated symptoms. Schizophrenia and other psychotic disorders are increasingly thought of as neurodevelopmental disorders, where multiple hits accumulate during critical periods of central nervous system (CNS) development to cause the disorders. The majority of patients with schizophrenia began with a prodromal phase characterized by subclinical symptoms of the disorder, which we will refer to hereafter as a state of clinical high risk for psychosis (CHR) [1,2,3,4]. In some studies, 22% of those meeting the criteria for CHR convert to a psychotic disorder at one year follow-up, as compared to 0.015% in the general population [5].

**Figure 1:**
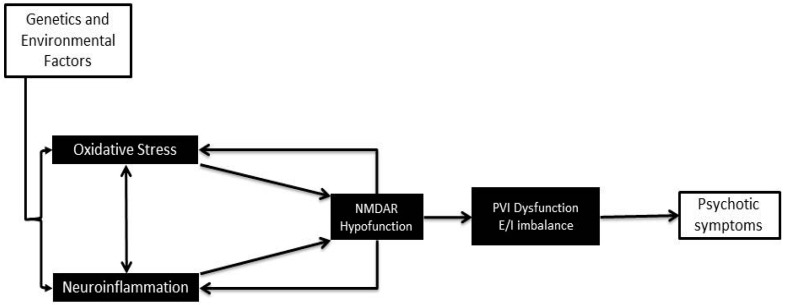
A simplified model of the link between neuroinflammation, oxidative stress and psychosis.

A growing body of literature supports a role for neuroinflammation and oxidative stress in the pathophysiology of psychosis. Although the underlying connection between these factors and development of psychosis is not yet clear, a promising target for these factors is a population of *N*-methyl-d-aspartate receptor (NMDAR)-containing parvalbumin interneurons (PVI) in the prefrontal cortex and hippocampus, which are likely disturbed during important developmental windows [6] (Figure 1).

In this review, we will propose a model suggesting how alterations in cellular homeostasis due to oxidative stress and immune dysfunction could lead to aberrant growth and/or pruning of these interneurons and lead to psychotic symptoms. We will then discuss efforts to characterize and intervene in the CHR population and discuss the implications of inflammation and oxidative stress to current and future therapies to try and prevent disease progression.

## 2. NMDAR Hypofunction and Psychosis

A major hypothesis about the origin of schizophrenia symptoms, is that they result from dysfunction in parvalbumin interneurons (PVIs), particularly through hypofunction of *N*-methyl-d-aspartate receptors (NMDARs) [7]. PVIs are fast-spiking GABAergic neurons that are crucial in synchronizing the firing of populations of pyramidal neurons in the cortex and generate the gamma oscillations associated with many of the higher-order cognitive processes that are interrupted in schizophrenia, such as working memory [7]. The first findings implicating NMDARs were studies of NMDAR antagonists, such as phencyclidine and ketamine, showing that subclinical doses of these drugs could mimic positive and negative symptoms, as well as cognitive deficits in healthy subjects [8]. These findings were further supported by other causes of NDMAR disruption, such as blockade by autoimmune antibodies in NMDAR encephalitis, which leads to severe psychosis and cognitive deficits [9,10]. In addition, NMDAR disruption may be upstream of the well-characterized hyperactivity of dopamine (D_2_) receptors in mesolimbic and mesocortical projections [11]. Whether cortical PVI disruptions are linked to mesolimbic dopaminergic dysfunction is not entirely clear, however, multiple studies have proposed that PVI disruption may cause dopamine dysfunction through loss of pyramidal cell inhibition in the hippocampal subiculum [11]. This disinhibition of pyramidal neurons leads to stimulation of the nucleus accumbens, followed by inhibition in ventral pallidum, effectively “taking the brake off”: the ventral tegmental area (VTA) dopamine release [12,13]. Thus, NMDAR hypofunction in PVIs may be a central feature of psychosis.

In addition, the healthy functioning of antioxidant and inflammatory/anti-inflammatory pathways may have important effects on the development and healthy functioning of PVIs. Decreased synapse density on the downstream pyramidal neuron, as measured by dendritic spine density, is a plausible mechanism by which disruptions in healthy development may occur [14]. This may occur in part through pathologically decreased perinatal growth or increased peripubertal pruning of these connections [7]. More precisely, since NMDARs play a key role in mediating long-term potentiation and other synaptic modifications that are dependent on the level of neuronal activity, low activity due to inflammation or oxidative stress could lead to long-term changes in PVIs, especially given that the relative number of NMDA receptors is highest perinatally, presumably because they play a critical role in deciding which neurons mature, and which are pruned [15]. In line with this, even mild antagonism of NMDARs in rodents postnatally can lead to lasting alterations in PVI number [16,17]. Moreover, PVIs are the last subset of interneurons to develop, which may explain how the effects of oxidative stress and inflammation (which are thought to be involved in the pathogenesis of many illnesses) may lead to the particular alterations seen in schizophrenia [7,18,19]. Thus, PVI NMDAR hypo-function seems to be an important factor in the development of psychosis.

## 3. Inflammation and Psychosis

Schizophrenia results from changes in the CNS that, at least for a significant subpopulation of patients, may result from neuroinflammation and abnormal immunological responses. Some of the first evidence for these effects came from epidemiological studies of the 1957 influenza pandemic showing a strong association between maternal infection in pregnancy and development of schizophrenia in offspring [20,21]. Subsequent studies demonstrated this effect to be dependent on immune activation and maternal cytokine release rather than the specific infectious agent [22,23]. Remarkably, it is suggested that as many as 14%–21% of schizophrenia cases could be prevented if maternal influenza infections were eliminated [24].

Another indicator of the importance of inflammation in schizophrenia comes from genetic studies. Multiple genome-wide association studies have implicated the major histocompatibility complex (MHC) island on chromosome 6, which is a cornerstone of the immune system, as having the strongest allelic association with schizophrenia [25,26]. In particular, the complement component 4 (*C4* gene) within the human leukocyte antigen (HLA) island was found to have a strong association with schizophrenia [26]. C4 is involved in both opsonization of pathogens, and in synaptic pruning, which may provide one connection to the developmentally timed nature of schizophrenia risk [26]. There are many more examples of genetic predisposition resulting from variation in inflammatory gene complexes—for example polymorphisms in some pro-inflammatory cytokine gene complexes (e.g., interleukin-1β, *IL1B*) may be linked to increased likelihood of developing schizophrenia [27]. These large-scale genetic and epidemiological studies lend a great deal of credence to the idea that inflammation is important in the pathogenesis of schizophrenia, but the details of how come primarily from preclinical studies.

### 3.1. Evidence from Preclinical Models

Preclinical animal models have provided supporting evidence for the role of the immune system in the development of psychosis, including the disruption of PVI development [28]. Much of this evidence comes from maternal immune activation (MIA) models used to study this process, which attempt to study the predisposition to schizophrenia resulting from prenatal infection of the mother. MIA models use a variety of immunogenic agents such as the viral mimic poly I:C (polyriboinosinic–polyribocytidylic acid) or the bacterial mimic lipopolysaccharide (LPS). MIA models have reliably produced anatomical and behavioural alterations in the pups of exposed rodents that are thought to be analogous to human psychosis [29,30]. In addition, multiple changes in key neurotransmitter systems characteristic of schizophrenia have been noted in the MIA model, including hippocampal NMDAR hypofunction and enhanced sensitivity to acute dopaminergic stimulation [31]. Immunologic changes, such as “priming” of the CNS’s resident macrophages (microglia) may be one way in which MIA predisposes the offspring to psychosis. Rodent studies using the MIA model have also shown that maternal infection can increase the number and activation of microglia in the brain of pups even once they reach adolescence [32,33]. Given that microglia are the resident macrophages of the brain, and are instrumental in the process of synaptic pruning by actively engulfing synaptic material, they may be implicated in the structural aberrancies that are thought to result from abnormal pruning in schizophrenia [34]. This may be how activated microglia affect NMDAR-containing synapses, and microglia may be partly responsible for the hypofunction observed in PVIs in schizophrenia [35,36].

### 3.2. Clinical Studies

Studies of patients with schizophrenia typically examine inflammatory status after the illness is already established [37,38]. However, in CHR individuals, numerous inflammatory cytokines and markers appear to predict conversion to psychosis even before the onset of a diagnosable illness [39]. Furthermore, increases in peripheral inflammatory markers have been observed in first episode psychosis, and are linked to severity of psychopathology and cognitive impairment [40,41]. There is also some evidence of a chronic inflammatory state in patients with schizophrenia, including reductions in anti-inflammatory cytokines, and increases in inflammatory ones [42,43]. Finally, treatment with antipsychotic medication may act, at least in part, through an anti-inflammatory mechanism by modifying cytokine levels [43,44].

In addition to surrogate blood markers and post-mortem studies, a number of imaging studies have attempted to elucidate the role of inflammation in psychosis pathogenesis. However, given the complexity of immune responses in the brain, imaging neuroinflammation in vivo has posed a significant challenge [45,46,47]. Currently, positron emission tomography (PET) imaging of mitochondrial 18 kDa translocator protein (TSPO), which measures microglial activation, is the most valid approach for studying neuroinflammation in vivo [48]. Of nine studies that investigated in vivo brain neuroinflammation in schizophrenia to date [46,48,49,50,51,52,53,54,55], four studies reported higher neuroinflammation in medicated schizophrenia patients as compared to healthy volunteers [50,51,53,55]. However, three out of these four studies used a first-generation radioligand for TSPO, [^11^C]PK11195, which is known to have important methodological limitations. Furthermore, the only study showing positive results using a second-generation TSPO radioligand used an alternative methodology, which was not replicated when using the gold standard in the field [55,56]. Consistent with this, recent studies using second-generation TSPO radioligand and the gold standard methodology showed no significant differences in neuroinflammation between medicated [49] or drug-naïve [48] first-episode psychosis or schizophrenia patients [46] compared to healthy volunteers. Taken together, in vivo PET studies on neuroinflammation in schizophrenia do not support increased microglial activation, as most of the PET studies that reported increased neuroinflammation in patients with schizophrenia or in individuals with CHR had methodological limitations, while studies using validated methodologies with larger sample sizes did not observe a significant group effect [48]. Thus, it seems that neuroinflammation might only be present in a subgroup of patients, or only present at very early stages of the disease, or simply that we cannot observe it using our current in vivo probes.

## 4. Oxidative Stress and Psychosis

Reactive oxygen species (ROS) are by-products of aerobic metabolism, produced primarily in the mitochondria of cells throughout the human body. These chemical species are strongly implicated in aging and a plethora of disease processes due to their ability to chemically alter cellular components such as lipids and proteins [57]. Under normal circumstances, endogenous antioxidants such as glutathione (GSH), neutralize these factors and protect human tissues from excess damage [58]. Without sufficient antioxidant levels to keep ROS under control, neurotoxicity can occur through oxidation of macromolecules such as DNA, proteins, and fats, and though the activation of cell signalling pathways that alter cell behaviour [59]. Over time, these changes might lead to some of the structural and functional alterations that are seen in psychosis [60].

### 4.1. Preclinical Studies

Animal studies have been instrumental in understanding the developmental effects of oxidative imbalances on NMDAR function in the development of psychosis. For example, depletion of GSH postnatally leads to lasting psychosis-like symptomatology [61]. Furthermore, GSH deficits can reduce NMDAR function, which may in turn lead to decreases in cortical PVI number [16,62]. Moreover, studies preventing the formation of GSH through genetic modification of its biosynthetic enzyme, glutamate cysteine ligase (GCL) show oxidative stress that precedes PVI deficits, leading to long-term prefrontal and hippocampal PVI abnormalities with loss of synchronicity in high-frequency firing, and prolonged plasticity of hippocampal PVIs [63,64,65]. Conversely, pharmacological replenishment of GSH with its precursor, *N*-acetylcysteine (NAC) prevents PVI dysfunction in multiple rodent models of psychosis [66,67]. The severity of damage to PVIs may be due to the very high metabolic demands of these fast-spiking neurons, which requires a robust antioxidant system [6,68]. One mechanism by which the brain appears to protect PVIs is through a feedback loop between NMDAR activity and GSH synthesis. Increases in NMDAR activation lead to increases in GCL activity, while changes in oxidative stress regulate NMDAR activation at the GRIN2A subunit [69,70,71]. In line with this, repeated NMDAR antagonism with ketamine in rodents has been shown to cause elevations in brain superoxide radicals with dysfunction of PVIs that were preventable by inhibition of NADPH oxidase [72]. All of this points to a close relationship between GSH and NMDARs in mitigating oxidative damage in the brain, particularly in vulnerable fast-spiking PVIs. Thus, the disruption of this feedback loop by deficiencies in NMDAR could foreseeably lead to uncontrolled oxidation and neurotoxicity to PVIs.

### 4.2. Clinical Studies

As compared to animal studies, the picture of oxidative stress in humans with psychosis is much less clear. Most studies examine markers of oxidative status in blood, such as glutathione (GSH), superoxide dismutase (SOD) and markers of lipid oxidation, while others look at cerebrospinal fluid (CSF), post-mortem tissue, and in general, a number of these markers indicate oxidative imbalance [58,73,74,75,76]. Moreover, there may be a relationship between decreases in blood GSH and psychosis symptoms, as well as alterations of brain volume in schizophrenia [77,78]. The results of recent clinical trials suggest that administration of NAC in humans, when used with antipsychotic medications, may alleviate negative and cognitive symptoms in patients with schizophrenia [79,80]. Gene and protein alterations related to oxidative stress have also been noted. For instance, familial variations in a subunit of GCL, the enzyme responsible for GSH synthesis may increase the risk of schizophrenia up to four-fold [81]. On the other hand, levels of oxidative markers appear to vary significantly with clinical status in cross-sectional studies [58]. A recent extensive review on studies on the relationship between schizophrenia and oxidative stress found that peripheral markers of GSH were consistently decreased, but found equivocal results for other antioxidants such as superoxide dismutase and catalase [82]. One explanation for these inconsistencies may be the heterogeneous nature of schizophrenia in terms of etiology, medication adherence, or episodic variations in disease severity among patients [83]. For this reason, it may be more useful to characterize pro- and anti-oxidant factors according to their role as state markers (i.e., symptom-correlated) and trait markers (i.e., symptom independent). Using this methodology, a recent meta-analysis found that total antioxidant status, as well as red blood cell (RBC) catalase and plasma nitrite appeared to be state markers of schizophrenia, while RBC superoxide dismutase appeared to be a trait marker for schizophrenia [58].

### 4.3. Connections between Inflammation and Oxidative Stress

Oxidative stress and inflammation are intricately linked, and many of the deleterious effects of oxidative stress are likely mediated by inflammation, and vice versa. At a molecular level, oxidative stress induces inflammation via activation of nuclear factor κB (NF-κB), a rapid-acting transcriptional activator of inflammatory response that can also induce the production of more free radicals [84,85,86]. Conversely, the immune system is a major source of oxidative stress because activated microglia use NADPH oxidase to generate reactive superoxide to destroy pathogens, which can also damage the brain’s own neurons if not properly balanced with antioxidants [87]. Furthermore, some studies show that the development of psychosis in immune activation models may be mediated by an imbalance between pro-oxidants and anti-oxidants [88,89]. For example, one study using a maternal immune activation (MIA) model showed elevations in multiple markers of oxidative stress in the hippocampus of male offspring, including a decreased ratio of GSH to its reduced form, glutathione disulfide (GSSG) that was reversible by NAC administration [89]. Taken together, it appears that immune activation and oxidative stress may have a close reciprocal relationship in the development of psychosis, though the mechanisms are not fully understood.

## 5. Clinical Implications

The mainstay in schizophrenia management, treatment with antipsychotics, is associated with several side effects [90] and does not ameliorate cognitive or negative symptoms, which are closely linked to functional outcome [91]. Furthermore, only about 40% of patients have good adherence to their medications at any given time [92,93].

According to the current consensus on management of psychosis, early intervention in psychosis is associated with better outcome [94]. In order to do this effectively, it will be necessary to implement a clinical staging model that includes flagging of patients based on the progression of disease (e.g., high risk or first-episode psychosis) [94]. Given the current evidence on the implications of oxidative stress and neuroinflammation in the pathogenesis of schizophrenia, there is a great deal of interest in intervening in these processes as early as possible to prevent onset or slow progression of the disorder.

In terms of identifying individuals early, a recent multi-centre trial attempted to distinguish CHR individuals’ likelihood of conversion based on blood samples [95]. They were able to isolate 17 markers that were predictive of conversion, including multiple markers of inflammation and oxidative stress [39]. In this study, the use of multiple markers likely contributed to the success of the blood panel. The use of a panel of markers, such as the 10 proposed by Flatow et al., to measure total antioxidant status (TAS), may further increase the predictive value of such prognostic tests [58]. Further to the goal of early identification, there was a recent push for a new diagnostic label of ‘attenuated psychosis syndrome’ in the Diagnostic and Statistical Manual, Fifth Edition (DSM-V) [96]. Though the proposal was deemed premature due to problems with reliability in clinical assessment, the diagnostic construct remains a major subject of research into disease progression-modifying treatments [96]. There have been some clinical trials in CHR populations using prophylactic therapies in an attempt to prevent the conversion to schizophrenia [97,98,99,100,101,102,103,104,105]. These have included the use of omega-3 polyunsaturated fatty acids (PUFAs), anti-psychotic use, and numerous psychosocial and cognitive behavioural interventions. None of the studies using anti-psychotics found a significant difference in conversion rates, but treatment with PUFAs appeared to have a substantial effect on conversion despite lack of efficacy in full-blown psychosis, with a relative risk of 0.18 [106]. The success of PUFA treatment is very promising given its excellent tolerability, and led to reductions in conversion, better functioning and a general decrease in psychiatric morbidity at 6.7 years follow-up [107]. The mechanisms theorized to be at play in the use of PUFAs include anti-oxidant effect, enhanced mitochondrial performance, and protection of myelin sheath integrity [108,109,110], which have not been directly tested (i.e., no target engagement studies to date). Thus, the success of the trial with PUFAs provides a promising piece of evidence that intervening in inflammatory or oxidative processes early could reduce or prevent transition to psychosis and perhaps alter disease course.

However, these results must be interpreted with caution. The clinical trial on prophylactic PUFA administration [106,111] was not replicated in a very recent multicenter randomized clinical trial showing no significant effect of PUFA on conversion rate of individuals at ultra-high-risk for psychosis [105]. Thus, although inflammation and oxidative stress appear to be involved in developmental predisposition to schizophrenia, there are conflicting results on their involvement in the actual transition to psychosis. While some peripheral markers of inflammation appear to be elevated, recent brain imaging studies have shown no difference between CHR individuals and controls [46,48]. Furthermore, we were not able to find any published papers imaging in vivo glutathione levels in CHR individuals. One explanation for the lack of activated microglia in CHR may be that microglia do not require activation in order to undertake synaptic pruning [112]. In addition, contradictory or ambiguous results may result in part from heterogeneity in the pathogenesis and biochemical profile of different psychosis subgroups. For example, a recent landmark paper showed evidence of a correlation between inflammatory markers in schizophrenia and specific structural and functional alterations, likely reflecting an etiologic subgroup of patients [113]. This may point towards the need for a staging system based not only on clinical symptomatology, but also on underlying pathology and endophenotypes [114]. It is also worth noting that psychosocial interventions have shown neutral to positive results across multiple centres and trials, and as such are currently considered first-line treatment in CHR due to low side effects, although the underlying mechanism by which these interventions work is currently unknown [106].

## 6. Conclusions

Recent strides have been made in our understanding of schizophrenia as a disease, including the adoption of the NMDAR hypofunction hypothesis and a rapidly increasing interest in characterizing people in early stages of psychotic disorders. It is increasingly evident that oxidative balance and neuroinflammation interact with the developing brain during perinatal and peripubertal critical periods, especially relating to the formation and pruning of specific synapses formed by PVIs. How exactly this process works at a molecular level remains hazy at best, although it may involve dysfunctional microglia-mediated pruning, disruption of the feedback loop between oxidative stress and NMDAR, and loss of pyramidal dendritic spine density.

As preclinical trials continue to elucidate the complex inflammatory and oxidative processes that lead to the development of psychosis-like phenotypes, there must be a simultaneous push to understand the disease process in humans. Unfortunately, anti-psychotics are only effective for positive symptoms while negative and cognitive symptoms have the greatest contribution to functional outcome and quality of life of psychotic patients. More high-quality clinical trials that test for target engagement with large sample sizes on antioxidant and anti-inflammatory treatments are needed, along with research like the study by Perkins and colleagues [39], looking into prognostic and diagnostic indicators for psychosis. It is noteworthy that due to the complexity of oxidative stress and inflammatory pathways in the human body, as well as the off-target effects that medications could potentially have, future clinical trials ideally need to be more specific in terms of what they are targeting in the human body. This must be paired with more basic research using techniques such as PET and MRS that provide in vivo information about the inflammatory and oxidative processes that are occurring in high-risk individuals.
